# Active packaging for Salmon stored at refrigerator with Polypropylene nanocomposites containing 4A zeolite, ZnO nanoparticles, and green tea extract

**DOI:** 10.1002/fsn3.1934

**Published:** 2020-10-19

**Authors:** Maryam Azizi‐Lalabadi, Leila Rafiei, Bahark Divband, Ali Ehsani

**Affiliations:** ^1^ Research Center for Environmental Determinants of Health (RCEDH) Kermanshah University of Medical Sciences Kermanshah Iran; ^2^ Department of Food Science and Technology Urmia University Faculty of Agriculture Urmia Iran; ^3^ Dental and Periodontal Research Center Tabriz University of Medical Sciences Tabriz Iran; ^4^ Inorganic Chemistry Department Faculty of Chemistry University of Tabriz Tabriz Iran; ^5^ Nutrition Research Center Department of Food Sciences and Technology Faculty of Nutrition and Food Sciences Tabriz University of Medical Sciences Tabriz Iran; ^6^ Food and Drug safety research center Tabriz University of medical science Tabriz Iran

**Keywords:** Nanocomposite, plant extract, synthetic polymer, Zeolite

## Abstract

In this study, three types of Polypropylene‐based (PP) films (two active nanocomposites and one control film) containing zinc oxide nanoparticles (ZnO NPs), 4A zeolite (4A Z), and green tea extract (GTE) were studied as modern active packaging's that can adjust the release of antimicrobial agents. The influence of PP nanocomposite with 3% (w/w) ZnO NPs/4A Z/GTE (treatment 1) and 6% (w/w) ZnO NPs/4A Z/GTE (treatment 2) on controlling microbial growth and preserving the sensory and chemical qualities of Salmon over nine days of storage at 4 ± 1°C was evaluated. The disk diffusion test revealed inhibition zones in the range of 10.98 ± 0.03 to 13.42 ± 0.01 m for treatments 1 and 2, respectively; the nanocomposite film with 6% ZnO NPs/4A Z/GTE had the highest antimicrobial effect against Gram‐negative bacteria (*p* < .05). Chemical analysis revealed that the initial peroxide value of Salmon was 0.68 ± 0.0 mEq/kg, which increased by day 9 to 12.3 ± 0.03 mEq/kg in the control sample, but rising only to 9.9 ± 0.01 and 7.3 ± 0.02 mEq/kg in treatments 1 and 2, respectively (*p* < .05). The shelf life of Salmon given treatment 2 increased significantly to nine days relative to the control. Accordingly, these nanocomposite films are promising as new active packaging for preventing microbial growth and preserving the quality of salmon.

## Graphical abstract

1. Zinc oxide nanoparticles and green tea extract have an effective antimicrobial property.
2. Zeolite as a support for nanoparticles can control the release of them and increase antimicrobial activities.
3. These Polypropylene nanocomposites can be considered as a new active packaging.

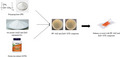

## 1 INTRODUCTION

Salmon fish is a type of seafood and valuable protein source, maintaining a significant role in the human diet. Salmon fish contain large amounts of important compounds, including fat‐soluble vitamins, trace elements, and polyunsaturated fatty acids (PUFA; mainly docosahexaenoic acid [DHA] and eicosapentaenoic acid [EPA]). The role of PUFA in brain cell growth during the embryonic period has been recognized (Hosseini et al., 2016). Also, PUFA can prevent heart disease, though high levels of PUFA in Salmon fish result in weak product quality. Fish spoilage leads to lipid oxidation and growth of microorganisms, as well as the consequent spread of unpleasant odor, color, taste, texture, and appearance; the nutritional value is also reduced (Hosseini et al., 2016). One of the common methods for preserving Salmon fish is the utilization of cold temperatures via refrigerators. Low temperatures slow down the speed of enzymatic reactions and microbial growth but do not completely inhibit them. Therefore, the innovation of new and modern way to preserve Salmon fish with minimal changes is essential (Alparslan & Baygar, 2017).

Given the increasing variety of food products and the heightened consumer tendency for use of fresh foods without any chemical preservatives, active packaging has been considered as a new technique in the food packaging industry. Active packaging is generally used to prevent the activity of microorganisms, enzymes and oxidative corruption (Biji et al., 2015). Utilization of preservatives such as inorganic antimicrobial materials (e.g., zinc oxide nanoparticles [ZnO NPs]) and plant extracts (e.g., green tea extract [GTE]) can be effective in improving the shelf life, quality, and safety of products without causing any side effects (Ates et al., 2013; Luo et al., 2015).

As a metal oxide, the importance of ZnO is highlighted through its low cost, high availability, nontoxicity, regular crystalline structure, high specific surface area, excellent thermo‐mechanical properties, UV‐barrier characteristics (Akbariazam et al., 2016; Kavitha et al., 2011; Meshram et al., 2017), and considerable antimicrobial activity certain viral, bacterial, and fungal species (Jesline et al., 2015; Luo et al., 2015). It should be noted that ZnO NPs under limited amount have been granted a “generally recognized as safe” (GRAS) status by the Food and Drug Administration (FDA, 2011).

Green tea plants, known as Camellia sinensis, are grown in China and northern India. Green tea leaf compounds include flavanols, flavonols, folic acid, phenolic compounds (e.g., catechins), caffeine, and theobromine. The most important catechin compounds are epicatechin, epicatechin gallate, epigallocatechin, and epigallocatechin gallate. Epigallocatechin gallate is the most abundant and active catechin with antioxidant activity in GTE (Akbari et al., 2019; Arezoo et al., 2020).

Recently, the application of nanoparticles and plant extracts in polymer matrices such as polypropylene (PP) has been looked at for the production of antimicrobial nanocomposite films that can maintain the quality, safety, and shelf life of food products (Lagaron et al., 2005). In fact, ZnO NPs and GTE inhibit bacterial growth by altering the permeability of the cell wall and membrane, activating reactive oxygen species (ROS), annihilating the protein network, and preventing DNA replication and enzymatic activities in bacteria (Kumar & Anthony, 2016; Maheswari et al., 2018; Sirelkhatim et al., 2015). Hence, with the aim of enhancing the quality of Salmon fish, this research concentrated on destroying bacteria or at least delaying their growth. To this end, the microbial, chemical and sensory properties of Salmon fish packaged in different PP nanocomposites were measured during storage at 4°C.

## 2 MATERIALS AND METHODS

### 2.1 Materials

Polypropylene (molecular weight: 0.85 g/cm^3^; melting point: 130 to 171°C; CAS number: 9003–07–0) were purchased from Arya polymer company, and zinc acetate dehydrate were obtained from Azar‐Kimia‐Khatam company, Tabriz, Iran. ZnO NPs were synthesized in the laboratory. 4A zeolite (4A Z) (Si/Al ≅ 2) was synthesized from clinoptilolite (clinoptilolite were purchased from Azar‐Kimia‐Khatam company). Dry leaves of green tea were purchased from Golestan Co. Methanol, ethanol, thiobarbituric acid (TBA), TBA reagent, magnesium oxide, hydrochloric acid, sulfuric acid, potassium iodide, starch, chloroform, sodium thiosulfate, phenolphthalein reagent, boric acid, sodium hydroxide, and diethyl ether were obtained from Merck. Micromedia supplied the medium cultures including Plate Count Agar (PCA) and de Man, Ragusa, Sharpe (MRS) agar. All reagents were of analytical grade.

#### 2.1.1 Bacterial stains

The Biological and Genetic Resources Center (Tehran, Iran) supplied all bacterial species including Escherichia coli O157:H7 (IBRC‐M 10698), Listeria monocytogenes (IBRC‐M 10671), Pseudomonas fluorescens (IBRC‐M 10752), and Staphylococcus aureus (IBRC‐M 10690).

### 2.2 Methods

#### 2.2.1 Determination of minimum inhibitory concentration (MIC) and minimum bactericidal concentration (MBC) of 4A Z/ZnO/GTE nanocomposites

The process of microdilution with 96‐well microplates was used to determine the MIC and MBC of the various samples of the study (Dhanasekar et al., 2018). Nutrient broth was used to culture the species of bacteria used (*E. coli* O157:H7, *L. monocytogenes*, *P. fluorescens*, and *S. aureus*) for 24 hr before adjusting them to 0.5 McFarland standard turbidity (1.5 × 10^8^ CFU/ml). Next, the test wells were filled with 160 μl of nutrient broth, before 20 μl bacterial suspensions and various concentrations (0.5, 1, 2, 3, 4, 5, 6, and 7 mg/ml) of 4A Z, ZnO NPs/4A Z, 4A Z/GTE, and ZnO NPs/4A Z/GTE dispersed with ultrasonic bath in distilled water were added. For confirmation, both positive (broth and ZnO NPs/4A Z/GTE) and negative controls (broth and each bacterial suspension) were included. Next, incubation (24 hr) of the microplates took place at 25°C for P. fluorescens and at 37°C for the other three bacterial species; a microplate shaker (Boeco) was applied for continuous shaking at 50–100 rpm during the incubation time. The minimal antibacterial substance concentration at which bacterial growth is not visible is defined as the MIC, while the lowest concentration required for destroying a specific bacterial species is regarded as the MBC. To distinguish between MIC and MBC, broth dilution tests involving subculturing to agar plates lacking the test agent were employed.

#### 2.2.2 Extraction of green tea extract (GTE)

Extraction was performed via the microwave method described by (Ghasemzadeh‐mohammadi et al., 2017). This method involved preparation of dried green tea leaves followed by extraction using clevenger. The dried leaves of green tea were completely crushed and sieved in order to increase the surface area of solvent exposed to dry matter and consequently enhance the extraction efficiency. In this procedure, 1 g of sieved particles was mixed with 100 ml of deionized water. Microwave extraction was performed for 30 min with 50 Hz frequency, 2,450 MHz output, 600 W output power, and 80°C maximum temperature. The mixture of green tea leaves and deionized water was poured into the tube at a ratio of 1/100 then thoroughly mixed. Finally, the extract was filtered through a vacuum filter before stored at 4°C.

#### 2.2.3 Preparation of the ZnO NPs/4A Z/GTE nanocomposite

The hydrothermal method was used to synthesize 4A Z (alumina silicates); the ratio of silica to alumina was approximately 2:1. As sources of silicon and aluminum, clinoptilolite and alumina were used and mixed with NaOH. Finally, the 4A Z nanocomposite was produced through the transfer of the mixture to a Teflon reactor set at 90°C (Khatamian et al., 2012). To prepare ZnO clusters, a mixture containing 0.17 g Zn (CH3CHOO) _2_.2H_2_O was mixed with 4A Z in a ratio of 5:95% (w/w) before being shaken for 30 min. Then, zinc acetate was integrated to 4A Z over 24 hr via the ion exchange process at 60°C. Next, the mixture was washed with distilled water before being dried at 80°C. The resulting powder was then calcined (2 hr; 500°C) to give the ZnO NPs/4A Z nanocomposite. In order to add GTE, 200 ml of GTE was added to 5 g of ZnO NPs/4A Z; the mixture was placed on a magnetic stirrer set at 40 rpm for 12 hr. Then, washing was done with distilled water before drying in the oven at 70°C.

#### 2.2.4 Preparation of polypropylene (PP) nanocomposite films with ZnO NPs/4A Z/GTE

Polypropylene films were prepared using the procedure described by Carlol et al, in 2013. This involved the use of a double helix extruder set at 180°C. Polypropylene powder (1 kg) with different concentrations of ZnO NPs/4A Z/GTE (3% and 6% in treatments 1 and 2, respectively) was added to the extruder (Alswat et al., 2016; Jiang et al., 2012; Khalaj et al., 2016; Lepot et al., 2011). These materials melted through the extruder in six heat zones and were thoroughly mixed by applying a variety of shearing and compressive forces. The melted material exited the extruder in strips before being inserted into the granulator. Finally, the granules were prepared by casting in the form of food packaging films (de Dicastillo et al., 2013).

#### 2.2.5 Evaluation of disk diffusion test of Polypropylene nanocomposite ZnO NPs/4A Z/GTE films

To evaluate the antibacterial impact of the prepared films, the disk diffusion assay was employed (Ehsani et al., 2016). After 18 hr of growth in nutrient broth, suspensions of P. fluorescens, S. aureus, L. monocytogenes, and E. coli O157:H7 bacteria were collected and adjusted to 0.5 McFarland standard turbidity (1.5 × 10^8^ CFU/ml) before being diluted in a ratio of 1:10 to achieve a density of bacteria of 1.5 × 10^6^ CFU/ml. In the next step, 10 mm slices of the nanocomposites were cut under sterile conditions and placed on Mueller‐Hinton agar; 0.1 ml bacterial suspensions (1.5 × 10^6^ CFU/ml) were then inoculated on the nanocomposite segments. To arrive at the optimum nanocomposite film for preserving salmon, the films were prepared in three separate groups, namely the bare PP film (control sample), PP/3% ZnO NPs/4A Z/GTE (treatment 1) and PP/6%ZnO NPs/4A Z/GTE (treatment 2). Incubation occurred for 24 hr at 37°C for all the species studied except P. fluorescens, which was incubated at 25°C. Then, a digital micrometer was used to measure the zone of inhibition surrounding the disks.

#### 2.2.6 Preparation of Salmon samples and treatments

Fresh Salmon were transferred to the laboratory immediately after being purchased at the Fish and Shrimp Market of Tabriz, Iran. After washing, aseptic conditions were maintained as the heads and tails were separated. Then, each 25 g samples of prepared fish were placed into three separate groups (control and treatments). Next, packaging of the control samples occurred within PP films, whereas the different PP nanocomposite films were utilized to pack the Salmon in the treatment groups (3% ZnO NPs/4A Z/GTE and 6% ZnO NPs/4A Z/GTE). Then, the packaged samples were refrigerated (4 ± 1°C) for 9 days; microbial and sensory evaluations took place initially and after 3, 6, and 9 days of storage.

#### 2.2.7 Microbial characterization

To conduct microbial analysis, a Stomacher 400 (Seward Medical, London, UK) was initially used to mix salmon (10 g) with 0.1% sterile peptone water (90 ml) for 1 min. Next, serial dilutions (from 10^–1^ to 10^–8^) were utilized to prepare solutions of different concentrations. Then, agar plates that had previously been prepared were inoculated with 100 μl of the diluted solutions. To determine the total viable count (TVC), incubation occurred in PCA for 48 hr at 37°C. To obtain the count of psychrotrophic bacteria, the temperature and duration of incubation on PCA used were 10°C and 7 days, respectively. In contrast, MRS agar was utilized for anaerobic incubation (25°C; 48 hr in anaerobic jar) ahead of determining the lactic acid bacteria count (Raeisi et al., 2015). All results were reported as log CFU/g sample.

#### 2.2.8 Determination of chemical properties of polypropylene nanocomposite films with ZnO NPs/4A Z/GTE

##### Evaluation of peroxide value (PV)

To measure the PV, 0.5 g of Salmon fish meat was mixed with 25 ml of acetic acid and chloroform (the ratio of acetic acid to chloroform was 3:2). Then, 1 ml of saturated potassium iodide was added to the mixture, which was kept in dark conditions for 10 min. In the next stage, 30 ml of distilled water and 1 ml of 1% starch solution were added to the mixture. Finally, titration by sodium thiosulfate was performed until the mixture be colorless (Heller et al., 2019). The PV was calculated using the following formula:

$$\text{Peroxide value}\left(\frac{\text{mEq}}{\text{kg}}\right)=\frac{V\times N\times 1,000}{W}$$

where "*V*" denotes the volume of consumed sodium thiosulfate, "*N*" denotes the normality of sodium thiosulfate, and "*W*" denotes the weight of the sample.

##### Evaluation of thiobarbituric acid (TBA)

The colorimetric method was used to measure TBA; 200 mg of the Salmon fish was transferred to a 25 ml specific container and then brought to volume using 1‐butanol. Next, 5 ml of TBA reagent was added to 5 ml of the prepared solution. In the next stage, the solution was placed in a hot water bath set at 95°C for 90 min before being put in an ice bath for 10 min. Finally, the absorbance was measured at 532 nm (Morsy et al., 2016). The TBA value was calculated via the following formula:

$$\text{TBA}\left(\text{mg}\right)=\frac{A_{s}‐A_{b}}{200}\times 50$$

where "*A*_s_" denotes the absorbance of each treatment and "*A*_b_" denotes the absorbance of the control sample.

##### Determination of total volatile basic nitrogen (TVB‐N)

Total volatile basic nitrogen values were determined via the modified Conway microdiffusion method with a Kjeltec 8400 (Foss, Sweden) (Cai et al., 2014). To this end, minced Salmon meat (10 g) was first added to distilled water (250 ml). Then, the mixture was transferred to a flask after the addition of 2 g of MgO and one drop of silicon. Next, this blend was distilled into a receiver flask containing 25 ml of 3% boric acid solution and an indicator (0.1 g of methyl red and 0.1 g of methylene blue in 100 ml of ethanol) before being titrated with 0.01 N HCl. Finally, the value of TVB‐N was obtained considering the volume of HCl consumed in mg/100 g of Salmon meat.

##### Determination of pH value

To measure the pH value of Salmon, 5 g of salmon was homogenized in 25 ml of distilled water for 30 s, and the resulting solution's pH was determined using a pH meter (pH 510; Eutech® CyberScan, Singapore) at room temperature according to AOAC regulations (AOAC, 2005).

#### 2.2.9 Sensory evaluation

To conduct the sensory evaluation, a panel of ten semitrained judges was asked to score the Salmon on a nine‐point hedonic scale in terms of odor, texture, color, and total acceptance in accordance with the method described by Jouki et al. (2014). The panelists were well educated regarding the characteristics of salmon as they were chosen from graduate students of the Department of Food Science and Technology of Tabriz University of Medical Sciences (Tabriz, Iran). The scale points were 1–4, 5–8, 9–11, and 12 for bad/unacceptable, good, very good, and excellent, respectively (Prabhakar et al., 2020).

#### 2.2.10 Statistical analysis

Statistical analyses were performed using SPSS software (version 21.0, IBM). All experiments were carried out in triplicates using different samples of salmon from the same batch on the same day and independent bacterial cultures. Analyses included the use of ANOVA, independent‐samples *t* test, and Tukey's post hoc test. Differences were considered significant at *p* < .05.

## 3 RESULTS AND DISCUSSION

### 3.1 MIC and MBC assessment of the 4A Z/ZnO NPs/GTE nanocomposites

The MIC and MBC of the 4A Z/ZnO NPs/GTE nanocomposites are demonstrated in Tables 1 and 2. The nanocomposites were active in the concentrations of 1–4 mg/ml versus *E. coli* O_157_:H_7_, *S. aureus*, *P. fluorescens*, and *L. Monocytogenes*. These results are confirmative of the results of Dhanasekar and Madhumitha et al. (Dhanasekar et al., 2018; Madhumitha et al., 2015). The optimal MICs were chosen for the ZnO NPs/4A Z, GTE/4A Z, and ZnO NPs/4A Z/GTE films as 2 ± 0.02, 3 ± 0.02, and 1 ± 0.01 mg/ml, respectively (Table 1); for MBC, the corresponding values were 3 ± 0.02, 3 ± 0.01, and 2 ± 0.01 mg/ml, respectively. It should be noted that in the negative control, we could see turbidity related to bacterial growth, whereas no turbidity was observed in the positive control.

**TABLE 1:** MIC of 4A Z/ZnO NPs/GTE PP nanocomposites against bacteria

	MIC (mg/mL)			
Bacteria	4A Z	ZnO,4A Z	GTE, 4A Z	ZnO,4A Z/GTE
*E. coli* O157:H7	–	2	3	1
*S. aureus*	–	3	4	2
*P. fluorescens*	–	1	2	2
*L. Monocytogenes*	–	2	3	3

Abbreviations: MIC, minimum inhibition concentration; PP, polypropylene; ZnO, Zinc oxide; 4A Z, 4A zeolite; GTE, green tea extract.

**TABLE 2:** MBC of 4A Z/ZnO NPs/GTE PP nanocomposites against bacteria

	MBC (mg/mL)			
Bacteria	4A Z	ZnO,4A Z	GTE, 4A Z	ZnO,4A Z/GTE
*E. coli* O157:H7	–	3	3	2
*S. aureus*	–	4	5	3
*P. fluorescens*	–	2	3	2
*L. Monocytogenes*	–	3	4	3

Abbreviations: MBC, minimum bactericidal concentration; PP, polypropylene; ZnO: Zinc oxide; 4A Z: 4A zeolite; GTE: green tea extract.

Gram‐negative bacteria were more susceptible to ZnO NPs and GTE than Gram‐positive bacteria. Gram‐positive bacteria have a thick peptidoglycan layer in their cell walls, which leads to greater resistance against nanoparticles and GTE compared to Gram‐negative bacteria. The cell wall of Gram‐negative bacteria has a very thin peptidoglycan layer, meaning that the entry of ZnO NPs and GTE to the cell is thus accelerated. The faster the particles enter the cell, the faster they react with the bacteria. Furthermore, these bacteria also have a negative charge in their lipopolysaccharide layer, which is significant in the incorporation of positive ions. This facilitates the entrance of ZnO NPs into the cell, which is followed by impairment in cell permeability as well as prevention of DNA replication and protein damage (Arezoo et al., 2020; Jalali et al., 2016; Mith et al., 2014; Sharma et al., 2016).

### 3.2 Disk diffusion assay of polypropylene polymer with 4A Z/ZnO NPs/GTE

The disk diffusion test was carried out to evaluate the antimicrobial effect of PP nanocomposites containing 4A Z/ZnO NPs/GTE (Table 3). The results showed that the nanocomposites with 6% ZnO NPs/4A Z/GTE had greater antimicrobial impact versus bacteria than those with 3% 4A Z/ZnO NPs/GTE. According to the results, Gram‐negative bacteria (particularly *E. coli* O_157_H_7_) were more sensitive to the ZnO NPs and GTE than Gram‐positive bacteria. Polypropylene nanocomposites without ZnO NPs and GTE had no impact on the mentioned bacteria. This was expectable as the main advantages of PP nanocomposites with 4A Z are improvements in physicomechanical properties, though these nanocomposites can also control the release of ZnO NPs and GTE, thereby enhancing the antimicrobial effect of the latter two agents against bacteria (Azizi‐Lalabadi et al., 2019). The highest zone of inhibition was seen against *E. coli* O_157_H_7_, measuring 12.73 ± 0.03 mm and 13.42 ± 0.01 mm for treatments 1and 2, respectively; *S. aureus* had the lowest inhibitory zone across all treatments (*p* < .05).

**TABLE 3:** Disk diffusion analysis of PP nanocomposites containing 4A Z/ZnO NPs/GTE against bacteria

	Diameter of inhibition zone (mm)		
Bacteria	Control sample	Treatment 1	Treatment 2
*E. coli* O157:H7	–	12.73 ± 0.03 a	13.42 ± 0.01 e
*S. aureus*	–	10.98 ± 0.03 b	11.09 ± 0.04 f
*P. fluorescens*	–	12.11 ± 0.04 c	13.09 ± 0.02 g
*L. Monocytogenes*	–	11.36 ± 0.01 d	11.86 ± 0.03 hr

Note

Control sample: Polypropylene (PP), Treatment 1: PP/3% ZnO NPs/4A Z/GTE and Treatment 2: PP/6% ZnO NPs/4A Z/GTE. 4A Z: 4A zeolite and GTE: green tea extract.

Data are presented as mean ± *SD* and analyzed with one‐way analysis of variance. Different letters in each day represent statistical significance among different treatments using the Tukey test.

It has been established that nanoparticles affect the permeability of the cell wall, consequently preventing processes such as DNA replication and protein synthesis (Luo et al., 2015). Reactive oxygen species (ROS) cause the death of bacteria by oxidizing phospholipids and thus reducing cellular energy (Ando et al., 2015; Jayaseelan et al., 2013). Our results are in agreement with those of other studies (Azizi‐Lalabadi et al., 2020; Slavin et al., 2017).

### 3.3 Microbial evaluation

In the present study, microbial alterations were assessed via total viable count (TVC), psychrophilic bacteria count, and lactic acid bacteria count in Salmon fish over 9 days of refrigerated storage.

#### 3.3.1 Total viable count

**FIGURE 1:**
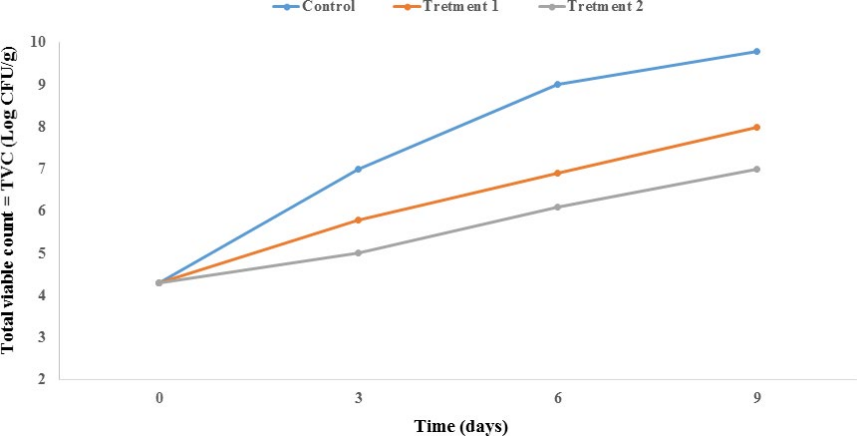
Effect of PP nanocomposite films containing ZnO NPs/4A Z/GTE on the total viable count (TVC) of Salmon during storage at 4°C. Each point represents the mean ± *SD*. Control sample: Polypropylene (PP), Treatment 1: PP/3% ZnO NPs/4A Z/GTE and Treatment 2: PP/6% ZnO NPs/4A Z/GTE. 4A z: 4A zeolite; GTE: green tea extract

Salmon, because of their high moisture content (above 70%), are a good source for growth of bacteria. The recommended acceptable TVC limit for salmon is 7 log CFU/g (Jouki et al., 2014). Based on Figure 1, the TVC for Salmon fish was initially 4.3 log CFU/g, which demonstrates that the quality of the salmon was acceptable (Soares et al., 2017). In all of the studied samples, the microbial load increased over time. However, it is notable that the microbial load remained within the acceptable range up until day nine of treatment with 6% ZnO NPs/4A Z/GTE. The microbial load for treatment 2 on the sixth and ninth days was 6.1 and 7 log CFU/g, respectively. This result is in accordance with other reported TVC values for fish. Yıldız in 2017 found that the initial microbial load for different fish species was between 2 and 6 log CFU/g (Yıldız, 2017). Ehsani et al. in 2020 reported that the TVC in fish during seven days of refrigerated storage was more than 7 log CFU/g (Ehsani et al., 2020). Such amounts were observed in the present study on the sixth day for 3% ZnO NPs/4A Z/GTE and on the ninth day for 6% ZnO NPs/4A Z/GTE, meaning that the latter nanocomposite showed higher antimicrobial properties. Yang et al. in 2008 demonstrated that green tea catechins (as polyphenolic compounds) have antimicrobial activity against both Gram‐negative and Gram‐positive bacteria (Yang et al., 2008). Irshad et al. in 2018 reported that GTE carries out antimicrobial activities via disturbing cell morphology, preventing bacterial enzyme function, inhibiting bacterial metabolism, and producing hydrogen peroxide through catechin oxidation (Irshad et al., 2018). Also, ZnO NPs exert their antimicrobial impact by producing free radicals on the surface of bacteria, thereby damaging the lipopolysaccharides of the cell membrane and releasing metal ions from the surface of the nanoparticles that bind to the cell membrane, consequently causing microbial cell death. In addition, indirect antimicrobial effects occur through the production and release of hydrogen peroxide; cellular destruction occurs due to the electrostatic interaction and direct penetration of ZnO NPs into the bacterial cell (Rojas et al., 2019).

#### 3.3.2 Psychrophilic bacteria count

**FIGURE 2:**
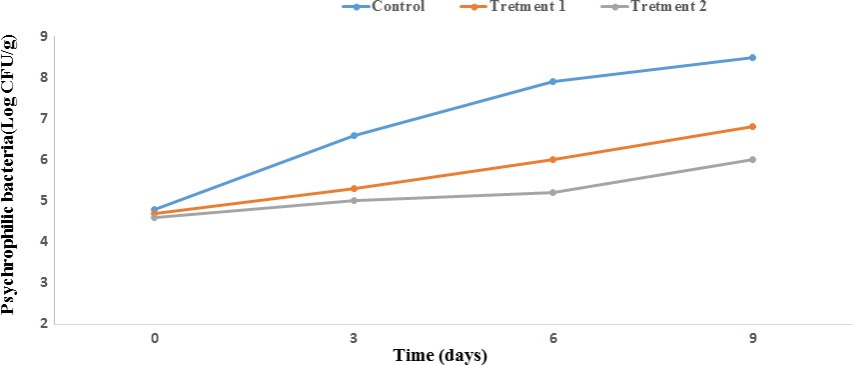
Effect of PP nanocomposite films containing ZnO NPs/4A Z/GTE on the psychrophilic bacteria count of Salmon during storage at 4°C. Each point represents the mean ± *SD*. Control sample: Polypropylene (PP), Treatment 1: PP/3% ZnO NPs/4A Z/GTE and Treatment 2: PP/6% ZnO NPs/4A Z/GTE. 4A Z: 4A zeolite; GTE: green tea extract

Seafood such as fish after fishing are mostly spoiled by psychrophilic bacteria such as Pseudomonas spp. because of their cold living conditions. These species are Gram‐positive and psychrotrophic bacteria, as obligate aerobes. Pseudomonas spp. are a key group of bacteria responsible for seafood spoilage (Jay et al., 2005). Our findings revealed that relative to the control film, the PP nanocomposite films had higher activity against psychrophilic bacteria (*p* < .05; Figure 2). The psychrophilic bacteria count was approximately 4.8 log CFU/g on the initial day of storage for all samples. By day 9, the bacterial population of the control sample had significantly increased to 8.5 log CFU/g, while such growth was not observed in treatments 1 and 2, which had counts of 6.8 and 6 log CFU/g, respectively. In fact, the highest increase was observed on the ninth day in the control sample, while the lowest bacteria count was on the ninth day in treatment 2. Our finding is in accordance with those of other researchers (Jayabalan et al., 2019; Polverari et al., 2019).

#### 3.3.3 Lactic acid bacteria count

**FIGURE 3:**
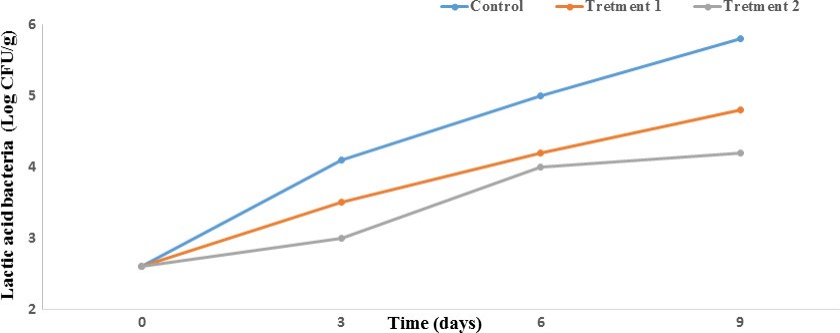
Effect of PP nanocomposite films containing ZnO NPs 4A Z and GTE on the lactic acid bacteria count of Salmon during storage at 4°C. Each point represents the mean ± *SD*. Control sample: Polypropylene (PP), Treatment 1: PP/3% ZnO NPs/4A Z/GTE and Treatment 2: PP/6% ZnO NPs/4A Z/GTE. 4A Z: 4A zeolite; GTE: green tea extract

The fact that the growth of lactic acid bacteria (LAB) is possible at low oxygen concentrations has resulted in the recognition of these bacteria as facultative anaerobes. Interestingly enough, a considerable proportion of the naturally occurring microflora in seafood is comprised of LAB (Aymerich et al., 2019). Initially, all samples had LAB counts of 2.6 log CFU/g; this amount increased during storage (Figure 3). Across the storage time, the nanocomposite films were able to significantly stunt the rise in LAB count (by approximately 1 log CFU/g) relative to the control (*p* < .05). The LAB count on the ninth day was 5.8, 4.8, and 4.2 log CFU/g for the control, treatment 1, and treatment 2, respectively. Our results are in accordance with those of a former study (Moosavi‐Nasab et al., 2019). In one study, it was declared that essential oil and plant extract significantly prevent LAB growth during storage of food products (Ankolekar et al., 2011).

### 3.4 Determination of chemical properties of polypropylene nanocomposite films containing ZnO NPs/4A Z/GTE

#### 3.4.1 Evaluation of peroxide value (PV)

**FIGURE 4:**
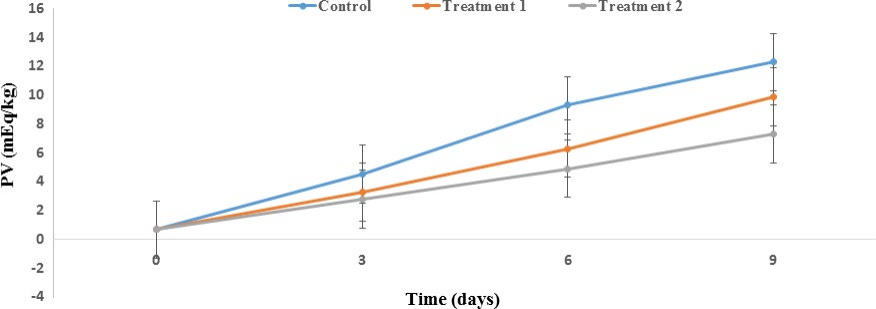
Effect of PP nanocomposite films containing ZnO NPs/4A Z/GTE on the peroxide value of Salmon during storage at 4°C. Control sample: Polypropylene (PP), Treatment 1: PP/3% ZnO NPs/4A Z/GTE and Treatment 2: PP/6% ZnO NPs/4A Z/GTE. 4A Z: 4A zeolite; GTE: green tea extract

The PV is an index of the primary products of fat oxidation. The PV values measured during refrigerated storage of the salmon are presented in Figure 4. The recommended acceptable limit of PV for salmon is 5 mEq/kg of fish (Jouki et al., 2014). Based on Figure 4, the initial PV of Salmon was 0.68 ± 0.0 mEq/kg, which increased to 12.3 ± 0.03, 9.9 ± 0.01, and 7.3 ± 0.02 mEq/kg on the ninth day of storage for the control sample and treatments 1 and 2, respectively. It is known that unsaturated fatty acids (UFA) in seafood lead to large PV alterations (Shadman et al., 2017). The rise in PV after 6 day in Salmon during storage indicates spoilage and a rapid increase in rancidity. Lipolytic enzymes have an important impact on the UFA of salmon; the measurement of free fatty acids is thus a good indicator for evaluating the effect of these enzymes on Salmon (Kop et al., 2019). In this study, the PV increased significantly across all samples after nine days of storage (*p* < .05). It should be noted that this trend developed at a higher rate in the control sample than either treatment. The lowest PV was observed in treatment 2, which could be attributed to the lower oxygen permeability and the moisture absorption properties of the 4A Z. In addition, GTE exerts its antioxidant effects by inhibition of chain reactions and degradation of hydroperoxides and free radicals (Raeisi et al., 2015). Our results are in agreement with those of Raeisi et al. (2015). Indeed, the results of this study indicate the effect of low temperature and the ZnO NPs/4A Z/GTE PP nanocomposite on PV regulation in salmon.

#### 3.4.2 Evaluation of thiobarbituric acid (TBA)

**FIGURE 5:**
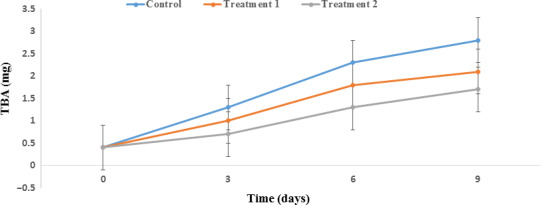
Effect of PP nanocomposite films containing ZnO NPs/4A Z/GTE on the thiobarbituric acid of Salmon during storage at 4°C. Control sample: Polypropylene (PP), Treatment 1: PP/3% ZnO NPs/4A Z/GTE and Treatment 2: PP/6% ZnO NPs/4A Z/GTE. 4A Z: 4A zeolite; GTE: green tea extract

The measurement of TBA is a good indicator for determining the progress of lipid oxidation and carbonyl and aldehyde production. The increase in the amount of TBA during storage may be due to the increase in free iron and other peroxidants in fish tissue (Raeisi et al., 2015). Figure 5 shows the effect of storage on TBA. Based on Figure 5, the amount of TBA increased significantly during storage (*p* < .05). The highest amount for TBA was observed on the ninth day in the control, while treatment 2 maintained the lowest TBA amount. The mechanism through which GTE decreases the TBA amount in active Salmon packaging is related to the ability of GTE in chelating iron ions from salmon proteins. The PP nanocomposite containing 6% ZnO NPs/4A Z/GTE had the most protective effect against fat oxidation in the salmon fillets due to less oxygen penetration to the salmon as well as greater diffusion of GTE from the surface of the film into the salmon. In fact, GTE, with its antioxidant activity, was able to suppress free radicals or slow down their production (Castro et al., 2019). Our results are in accordance with those of Raeisi et al. (2015) and Castro et al. (2019).

#### 3.4.3 Determination of total volatile basic nitrogen (TVB‐N)

**FIGURE 6:**
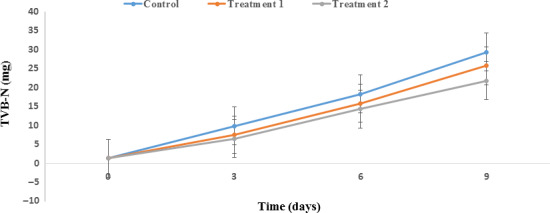
Effect of PP nanocomposite films containing ZnO NPs/4A Z/GTE on the total volatile basic nitrogen of Salmon during storage at 4°C. Control sample: Polypropylene (PP); Treatment 1: PP/3% ZnO NPs/4A Z/GTE; Treatment 2: PP/6% ZnO NPs/4A Z/GTE. 4A Z: 4A zeolite; GTE: green tea extract

The TVB‐*N* value has an important role in the quality assessment of salmon. This index includes the amount of ammonia and volatile amines as major indicators of degradation and spoilage of Salmon proteins. When TVB‐N reaches 25 mg per 100 g of Salmon, spoilage is identified (Jouki et al., 2014; Volpe et al., 2015). The results of evaluation of TVB‐N in Salmon during refrigerated storage are shown in Figure 6. The initial amount of TVB‐N was 1.3 ± 0.0 mg, increasing after nine days of storage to 29.3 ± 0.01, 25.7 ± 0.03, and 21.8 ± 0.02 mg in the control sample and treatments 1 and 2, respectively. After six days of storage, the amount of TVB‐N remained below the permitted limit across all samples. Also, it should be noted that the amount of TVB‐N in treatment 2 remained below the permitted limit after nine days of storage. The highest amount of TVB‐N was found in the control sample, while the lowest increase in TVB‐N was observed in treatment 2 (*p* < .05). Our findings are in accordance with those of other researchers (Ojagh et al., 2010; Shadman et al., 2017). Reasons for these differences in TVB‐N include the enhanced microbial load or the ability of the plant extract to de‐aminate nonprotein nitrogen compounds (Ojagh et al., 2010). Feng et al. in 2017 evaluated the effect of the polyphenols of GTE on the amount of TVB‐N during storage of Salmon. They confirmed that TVB‐N increased during Salmon storage; however, the antioxidant activity of the polyphenols of GTE resulted in the increase to be much higher in the control sample than the treatments (Feng et al., 2017).

#### 3.4.4 Determination of pH value

**FIGURE 7:**
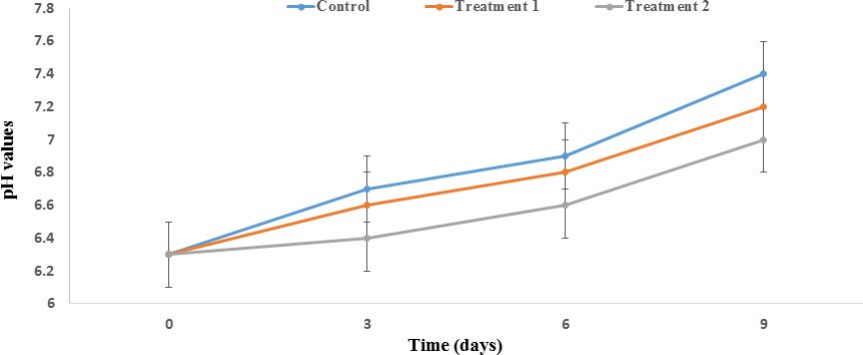
Effect of PP nanocomposite films containing ZnO NPs/4A Z/GTE on the pH value of Salmon during storage at 4°C. Control sample: Polypropylene (PP); Treatment 1: PP/3% ZnO NPs/4A Z/GTE; Treatment 2: PP/6% ZnO NPs/4A Z/GTE. 4A Z: 4A zeolite and GTE: green tea extract

The pH is an important factor that changes during Salmon storage. The pH value increased significantly during the storage of Salmon. The initial pH value was 6.3 ± 0.0 which increased after nine days of storage to 7.4 ± 0.02, 7.2 ± 0.02, and 7 ± 0.00 in the control sample and treatments 1 and 2, respectively (Figure 7). The reason for such rises in pH is the formation of nitrogenous compounds such as ammonium and biogenic amines as a result of enzymatic activity as well as the proteolytic activity of psychrophilic bacteria (Lee et al., 2019).

### 3.5 Sensory evaluation

Table 4 presents the results obtained from the evaluation of the Salmon samples in terms of sensory properties. Despite the fact that the Salmon samples all had acceptable sensory properties on the first day, significant drops in sensory features were found over the storage time (*p* < .05; Prabhakar et al., 2020). The control sample maintained good sensory characteristics until the third storage day, though acceptable texture, color, odor, and total acceptance scores were recorded even after nine days of storage in both treatments. Among the treatments, treatment 1 received a lower score compared with treatment 2 in terms of sensory evaluation. In fact, the higher total acceptance of treatment 2 indicates the ZnO NPs‐GTE interaction induced strong antimicrobial activity, preventing undesired alterations in texture, odor, and color. Significant differences were found between the control and treatment samples for total acceptance at days 3, 6, and 9 of refrigerated storage. These results are consistent with those obtained in related studies (Azizi‐Lalabadi et al., 2020; Shahbazi & Shavisi, 2018; Yuan et al., 2016). Indeed, the progressive release of ZnO NPs and GTE from the PP nanocomposite matrix exerted preservative impacts on sensory characteristics including texture, smell, and total acceptance.

**TABLE 4:** Effect of polypropylene nanocomposite films containing 4A Z/ZnO NPs/GTE on the sensory evaluation of Salmon at 4°C

Days	Groups	Color Mean ± *SD*	Odor Mean ± *SD*	Texture Mean ± *SD*	Total acceptance Mean ± *SD*
0	Control	12.00 ± 0.00	12.00 ± 0.00	12.00 ± 0.00	12.00 ± 0.00
3	Control Treatment 1 Treatment 2	8.34 ± 0.05 a 9.13 ± 0.04 b 10.21 ± 0.03 c	8.25 ± 0.03 a_1_ 11.19 ± 0.03 b_1_ 11.5 ± 0.05 c_1_	10.5 ± 0.08 a_2_ 11.5 ± 0.04 b_2_ 11.5 ± 0.04 c_2_	10 ± 0.04 a_3_ 11 ± 0.07 b_3_ 11 ± 0.08 c_3_
6	Control Treatment 1 Treatment 2	5.40 ± 0.06 a 6.25 ± 0.04 b 8.05 ± 0.05 c	6.13 ± 0.03 a_1_ 9.28 ± 0.03 b_1_ 9.5 ± 0.03 c_1_	7.5 ± 0.03 a_2_ 9 ± 0.04 b_2_ 9 ± 0.06 c_2_	8 ± 0.05 a_3_ 9 ± 0.04 b_3_ 10 ± 0.06 c_3_
9	Control Treatment 1 Treatment 2	2. 5 ± 0.05 a 4 ± 0.05 b 6.5 ± 0.06 c	4 ± 0.04 a_1_ 7 ± 0.04 b_1_ 7 ± 0.06 c_1_	5 ± 0.05 a_2_ 7.5 ± 0.2 b_2_ 7.5 ± 0.08 c_2_	6. 5 ± 0.04 a_3_ 7.5 ± 0.1 b_3_ 8 ± 0.04 b_3_

Note

Control sample: Polypropylene (PP), Treatment 1: PP/3% ZnO NPs/4A Z/GTE and Treatment 2: PP/6% ZnO NPs/4A Z/GTE. 4A Z: 4A zeolite and GTE: green tea extract.

Data are presented as mean ± *SD* and analyzed with one‐way analysis of variance. Different letters in each day represent statistical significance among different treatments using the Tukey.

## 4 CONCLUSION

In this study, PP nanocomposite films including 4A Z/ZnO NPs/GTE were used to increase the shelf life of Salmon. The results showed that the nanocomposites significantly increase the shelf life of Salmon (*p* < .05). Furthermore, the PV, TBA, and TVB‐N indices of Salmon all increased significantly during storage (*p* < .05). Sensory evaluation revealed that the texture, color, odor, and general acceptance of the Salmon samples in the treatment groups were significantly higher relative to the control (*p* < .05). The results of microbial analysis showed that the TVC, psychrophilic bacteria count, and lactic acid bacteria count all increased during Salmon storage, but these trends were significantly lower in the treatments compared to the control (*p* < .05). In conclusion, producing PP nanocomposite packaging's containing ZnO NPs/4A Z/GTE is an effective, promising and efficient method for increasing the shelf life, quality, and safety of salmon.

## CONFLICTS OF INTEREST

All author declares that there is no conflict of interest.

## AUTHOR'S CONTRIBUTION

Maryam Azizi‐Lalabadi write the manuscript, Leila Rafiei do experiments, Bahark Divband involved in data analysis, and Ali Ehsani designed the work.

## ETHICAL APPROVAL

This article does not contain any studies with human participants or animals performed by any of the authors.
